# Cas7 meets Cas14: a strategic partnership in the type VII CRISPR-Cas

**DOI:** 10.1093/procel/pwae056

**Published:** 2024-10-21

**Authors:** Yao Liu, Senfeng Zhang, Chunyi Hu

**Affiliations:** Department of Biological Sciences, Faculty of Science, National University of Singapore, Singapore 117543, Singapore; Department of Biological Sciences, Faculty of Science, National University of Singapore, Singapore 117543, Singapore; Department of Biological Sciences, Faculty of Science, National University of Singapore, Singapore 117543, Singapore; Department of Biochemistry, Yong Loo Lin School of Medicine, National University of Singapore, Singapore 117543, Singapore; Precision Medicine Translational Research Programme (TRP), National University of Singapore, Singapore 117597, Singapore

**Figure 1.**
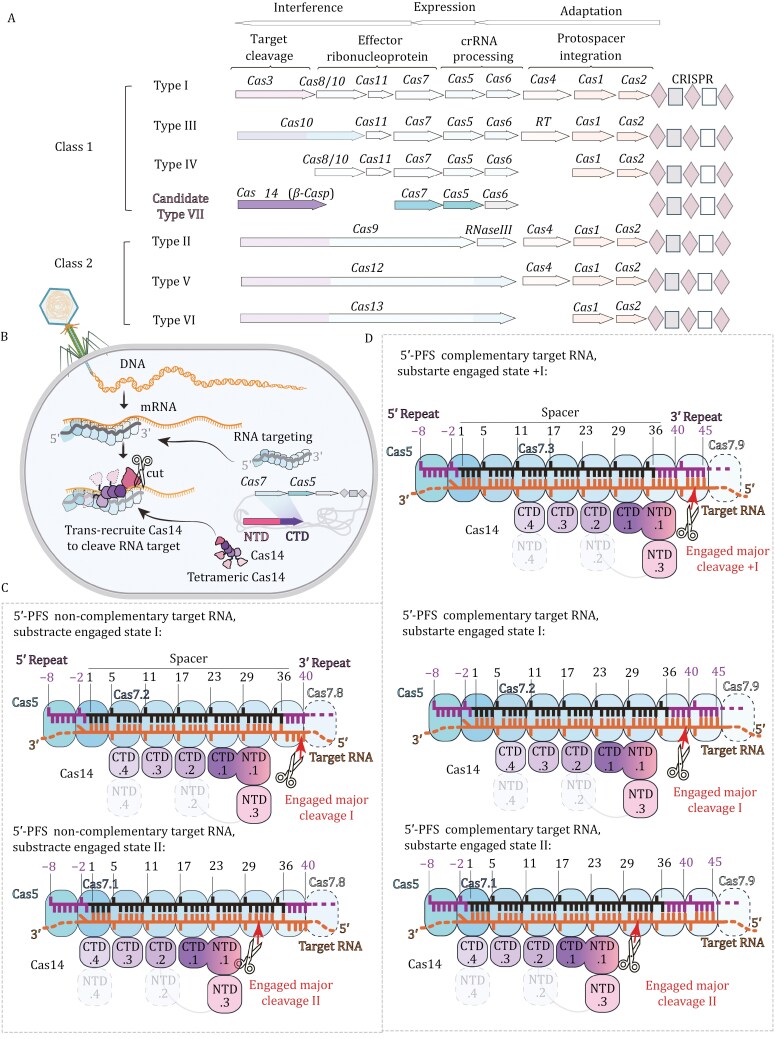
Schematic representation and mechanism Insights into the candidate type VII CRISPR-Cas system. (A) Classification and functional overview of CRISPR-Cas systems: The CRISPR-Cas systems are divided into two major classes based on their effector protein composition. Class 1 systems (types I, III, and IV) use multi-subunit complexes, while Class 2 systems (types II, V, and VI) rely on a single protein effector. The newly identified type VII system, a candidate member of Class 1, exhibits a unique architecture involving a CRISPR RNA (crRNA)-guided ribonucleoprotein complex formed by Cas5 and Cas7 proteins. Unlike other systems, the type VII CRISPR-Cas utilizes Cas14, a distinct nuclease, for RNA cleavage. (B) Functional and mechanistic schematic of the type VII interference complex. The crRNA-Cas5-Cas7 complex identifies, and binds target RNA through complementary base pairing with the crRNA (guide RNA), forming a functional interference complex. Cas14 is then recruited in a manner dependent on the presence of target RNA, where it executes precise cleavage of the RNA, thereby enabling the bacterial host to effectively defend against phage attacks. (C) Substrate-engaged states of the type VII CRISPR-Cas complex on 5ʹ-PFS non-complementary RNA target (CTR). The Cas5–Cas7-crRNA complex engages with the CTR, forming a substrate-engaged state. Tetrameric Cas14 associates with this complex in two distinct configurations, each aligned with a specific cleavage site on the target RNA. These configurations, termed states I and II, position Cas14 differently along the RNA, resulting in stepwise cleavage of the substrate. (D) Substrate-engaged states of the type VII CRISPR-Cas Complex on 5ʹ-PFS complementary RNA target (NTR). This figure depicts how Cas14 processes the NTR. The extended Cas5-Cas7 filament enables Cas14 to bind at three distinct sites on the RNA, leading to the generation of three major cleavage products labeled as + I, I, and II. Cas14 likely disassembles from the interference complex, and the cleavage products, such as the major cleavage I product, may act as substrates for subsequent rounds of cleavage. The 5ʹ-end PFS at the target RNA influences the cleavage pattern, which differs from the type III system where the 3ʹ-end PFS has an impact.

CRISPR (clustered regularly interspaced short palindromic repeats)-Cas systems, fundamental to prokaryotic adaptive immunity, are divided into two major classes based on their effector protein composition. Class 1 systems, including types I, III, and IV, utilize multi-subunit complexes to carry out interference (Wang et al., 2022), while Class 2 systems, encompassing types II, V, and VI, rely on single protein effectors to accomplish the same defence task (Dong et al., 2016, 2017; Makarova et al., 2020). The candidate type VII CRISPR-Cas system, a recent addition to Class 1, features a ribonucleoprotein complex composed of Cas5 and Cas7 proteins, guided by CRISPR RNA (crRNA) (Altae-Tran et al., 2023) (Fig. 1A). However, what truly sets this system apart is the recruitment of Cas14, a distinct nuclease with a β-CASP (CPSF–Artemis–SNM1–Pso2) domain homologous to RNase J, which executes RNA cleavage—a function typically performed by other effectors in different CRISPR systems.

In their study, Yang et al. (2024) systematically dissected the structural and functional components of the type VII CRISPR-Cas system. They began by utilizing cryo-electron microscopy to resolve seven distinct structures of the Cas14-bound interference complex, capturing it in various functional states. These structures revealed that the type VII system utilizes a ribonucleoprotein complex formed by Cas5 and Cas7 proteins, guided by CRISPR RNA (crRNA), to target RNA (Fig. 1B). Unlike in other type III CRISPR systems, where Cas7 or Cas11 are responsible for RNA cleavage (Guo et al., 2019; Hu et al., 2022; Huo et al., 2018; Liu et al., 2022; Wang et al., 2022; You et al., 2019), the researchers found that in the type VII system, Cas7’s nuclease site is inactive. Instead, a distinct nuclease, Cas14, is recruited to perform the RNA cleavage (Fig. 1B).

The researchers discovered that Cas14 is a tetrameric protein that is recruited to the Cas5–Cas7 complex in a target RNA-dependent manner (Fig. 1B). The N-terminal catalytic domain of Cas14 binds a stretch of the substrate RNA, enabling precise cleavage, while the C-terminal domain primarily anchors Cas14 to the Cas5–Cas7 complex. Through a series of biochemical cleavage assays, the researchers confirmed that Cas14 binds to different sites on the Cas5–Cas7 complex to execute individual cleavage events. They also revealed that a plugged-in arginine residue of Cas7, positioned within a C-shaped clamp of the C-terminal domain, precisely modulates Cas14 binding, ensuring controlled RNA cleavage.

The study offered deep insights into the substrate-engaged states of the type VII CRISPR-Cas complex. For the 5ʹ-protospacer flanking sequence (PFS) non-complementary RNA target, also known as the cognate target RNA (CTR), Cas14 interacts with the Cas5–Cas7 complex in two distinct configurations, referred to as states I and II. Each configuration corresponds to a specific cleavage site on the RNA, with Cas14 sequentially positioning itself along the RNA, leading to a stepwise cleavage of the substrate. Specifically, the state I cleavage site is at the +39th base of the target RNA, complementary to the crRNA spacer, while the State II cleavage site is at the +31st base of the crRNA spacer complementary target site (Fig. 1C). When the system encounters a 5ʹ-PFS complementary RNA target, or non-cognate target RNA (NTR), Cas14’s interaction with the extended Cas5–Cas7 filament becomes more complex. The filament provides three distinct binding sites for Cas14, resulting in the generation of three major cleavage products, corresponding to the 43rd, 39th, and 31st bases of the crRNA spacer complementary target site. This indicates that Cas14 can process the RNA in a highly regulated manner, influenced by the presence of a complementary PFS at the 5ʹ end of the target RNA (Fig. 1D). This is notably different from type III systems, where a complementary 3ʹ-PFS significantly impacts the cleavage process.

Despite these insights, several critical questions remain unanswered. For instance, without integrase proteins like Cas1 and Cas2, which are typically involved in the integration of CRISPR arrays, how does the type VII system integrate its CRISPR array? This is a puzzling question that warrants further investigation. Additionally, the potential applications of the type VII system are worth exploring. Without Cas14, the system requires only the Cas5 and Cas7 genes, making it highly compact and theoretically ideal for RNA targeting, RNA imaging, and even RNA editing when fused with RNA editors. On the other hand, with Cas14, the system could be used for targeted RNA degradation, offering versatile tools for RNA manipulation.

Future research should delve deeper into the unique advantages of the type VII CRISPR-Cas system in RNA targeting and explore how its compact nature could be harnessed to design more efficient genetic tools. The type VII system may open new avenues for RNA-related research and therapeutic approaches, such as developing tools for RNA degradation or creating compact gene editing systems, particularly in scenarios where high precision and minimal off-target effects are essential (Chen et al., 2020; Liu et al., 2023). As research progresses, the type VII CRISPR-Cas system stands out as a promising new tool for RNA-targeting applications, with potential impacts across various scientific and medical fields.
